# A novel homozygous *ARFGEF2* splice-site variant causing periventricular nodular heterotopia with microcephaly

**DOI:** 10.3389/fped.2026.1758158

**Published:** 2026-05-22

**Authors:** Xuefang Liu, Lingyu Pang, Jingjie Li, Jing Zhang, Wenjuan Wu, Xin Li, Yubing Gong, Yueying Dou, Fang Chen, Suzhen Sun

**Affiliations:** 1 First Department of Neurology, Hebei Children’s Hospital, Shijiazhuang, China; 2 First Department of Neurology, Hebei Provincial Clinical Research Center for Child Health and Disease, Shijiazhuang, China; 3 First Department of Neurology, Hebei Provincial Key Laboratory for Pediatric Epilepsy and Neurological Disorders, Shijiazhuang, China

**Keywords:** ARFGEF2 gene, microcephaly, minigene splicing assay, periventricular nodular heterotopia, West syndrome

## Abstract

**Background:**

The *ARFGEF2* gene encodes the brefeldin A (BFA)-inhibited GEF2 protein (BIG2), which is distributed in the trans-Golgi network and plays a crucial role in neuronal proliferation and migration during cortical development through its regulation of vesicle transport. Pathogenic mutations in the *ARFGEF2* gene are associated with autosomal recessive periventricular nodular heterotopia with microcephaly (ARPHM). To date, only slightly more than 20 cases have been reported worldwide. Herein, we presented a case of a patient with West syndrome who was ultimately diagnosed with ARPHM caused by a homozygous variant in the *ARFGEF2* gene.

**Methods:**

To identify disease-causing mutations, we performed exome sequencing (ES) of a child with West syndrome, and subsequently employed a minigene splicing assay to evaluate the functional impact of the *ARFGEF2* gene splicing variant.

**Results:**

The patient's clinical manifestations, examination results, treatment, and follow-up course were comprehensively documented. ES revealed a homozygous NM_006420.3: c.5181+1G>T variant in the *ARFGEF2* gene. Subsequent functional analysis using a minigene splicing assay confirmed that this variant disrupts normal mRNA splicing, causing complete exon 38 skipping and a 118-bp deletion. The translation of this aberrant transcript is predicted to induce a frameshift, resulting in a truncated protein (p.Val1689SerfsTer20).

**Conclusion:**

A novel pathogenic variant was identified by ES, and a minigene splicing assay established its disruptive impact on *ARFGEF2* mRNA splicing. This study expands the genetic spectrum of *ARFGEF2* and provides laboratory evidence for clinical diagnosis.

## 1 Introduction

Periventricular nodular heterotopia (PNH) is a common form of cortical malformation caused by impaired neuronal migration during embryonic development, resulting in abnormal aggregation of gray matter cells around the ventricles rather than their proper migration to the cerebral cortex. PNH is a form of gray matter heterotopia associated with drug-resistant epilepsy and developmental delay. Variants in the X-linked *FLNA* gene (MIM 300017) are the most common monogenic cause of PNH. However, rare biallelic variants in The ADP-ribosylation factor guanine nucleotide-exchange factor-2 (*ARFGEF2*, MIM605371) can also lead to PNH (1, 2). Sheen (3) described autosomal recessive periventricular nodular heterotopia with microcephaly (ARPHM, MIM608097) caused by homozygous mutations in the *ARFGEF2* gene. They reported that severe developmental delay and recurrent infections were the main clinical features, with no major extra-neurological abnormalities observed. Brain magnetic resonance imaging revealed PNH. Brefeldin A (BFA)-inhibited GEF2 protein (BIG2), encoded by *ARFGEF2*, is primarily distributed in the trans-Golgi network (TGN) and involved in neurodevelopment by regulating vesicular trafficking from the Golgi apparatus. Biallelic pathogenic variants in *ARFGEF2* can lead to PNH through an autosomal recessive inheritance pattern.

ARPHM caused by pathogenic variants in *ARFGEF2* is extremely rare, with only a few cases reported to date. Herein, we present a case of ARPHM caused by a homozygous variant in the *ARFGEF2* gene (NM_006420.3: c.5181+1G>T) which has never been reported before. The patient exhibited severe global developmental delay, refractory epilepsy, dystonia, and microcephaly. We describe the clinical characteristics of the patient, the pathogenicity of the genetic variant, and the results of genetic testing.

## 2 Materials and methods

### 2.1 Clinical data: clinical information of the patient

In this study, a male pediatric patient with epilepsy was enrolled. A series of auxiliary examinations and developmental assessments were conducted for phenotypic evaluation, including video-electroencephalography (VEEG), brain magnetic resonance imaging (MRI), echocardiography, brainstem auditory evoked potentials, and the Gesell Developmental Schedules (GDS).

### 2.2 Genetic testing

The EDTA-treated peripheral blood were collected with informed consent of the patient and his parents. The genomic DNA was extracted using the Blood Genome Column Medium Extraction Kit (Kangweishiji, China) according to the manufactural instructions. The extracted DNA samples were subjected to quality controlling using Qubit 2.0 fluorimeter and electrophoresis with 1% agarose gel for subsequent experiments. Protein-coding exome enrichment was performed using xGen Exome Research Panel v2.0 (IDT, Iowa, USA), which targets 39 Mb protein-coding region (19,396 genes) of the human genome and covers 51 Mb of end-to-end tiled probe space. High-throughput sequencing was performed by MGISEQ-T7 series sequencer, and not less than 99% of target sequence were sequenced. The sequencing process was performed by Beijing Chigene Translational Medicine Research Center Co., Ltd, 100875, Beijing. Raw data were processed by fastp for adapters removing and low-quality reads filtering. The paired-end reads were performed using Burrows-Wheeler Aligner (BWA) to the Ensemble GRCh37/hg19 reference genome. Base quality score recalibration together with SNP and short indel calling was conducted using GATK. According to the sequence depth and variant quality, SNPs and Indels were screened so that high quality and reliable variants were obtained. The online system independently developed by Chigene (https://www.chigene.org) was used to annotate database-based minor allele frequencies (MAFs), and ACMG practice guideline-based pathogenicity of every yielded gene variant. In accordance with the ACMG guidelines for pathogenicity classification, OMIM, HGMD and ClinVar databases were used as references to evaluate the pathogenicity of each variant.

### 2.3 Minigene splicing assay

To validate the effect of the NM_006420.3: c.5181+1G>T mutation on the mRNA splicing of gene *ARFGEF2*, we performed a minigene splicing assay.Genomic DNA (gDNA) from -an individual who does not have the predicted splice site variant was amplified using primers designed to generate two fragments: one containing 346 bp from the upstream region and the other containing 450 bp from the downstream region of intron 37, with the central portion of the intron deleted. These two fragments were gel-purified and subsequently assembled via recombination into a pMini-CopGFP vector that had been linearized by digestion with *Bam*HI and *Xho*I restriction enzymes. The constructed plasmid was verified by Sanger sequencing, confirming the successful generation of the wild-type plasmid. Using this wild-type plasmid as a template, a mutant plasmid was generated by site-directed mutagenesis to introduce the NM_006420.3: c.5181+1G>T mutation. Successful construction of the mutant plasmid was also confirmed by Sanger sequencing. The constructed plasmids were transfected into 293T cells using Lipofectamine 2,000 transfection reagent. The 293T cells were cultured in DMEM medium supplemented with 10% fetal bovine serum (FBS) and 1% penicillin-streptomycin (P/S) at 37 °C in a humidified atmosphere of 5% CO₂. Total RNA was extracted from the transfected cells 24 to 48 h post-transfection using Trizol reagent. The cDNA was amplified by PCR using specific primers. The resulting PCR products were analyzed by agarose gel electrophoresis and subsequently subjected to Sanger sequencing. The splicing patterns were determined by analyzing the sequencing chromatograms.

## 3 Results

### 3.1 Clinical manifestations

**Figure 1:**
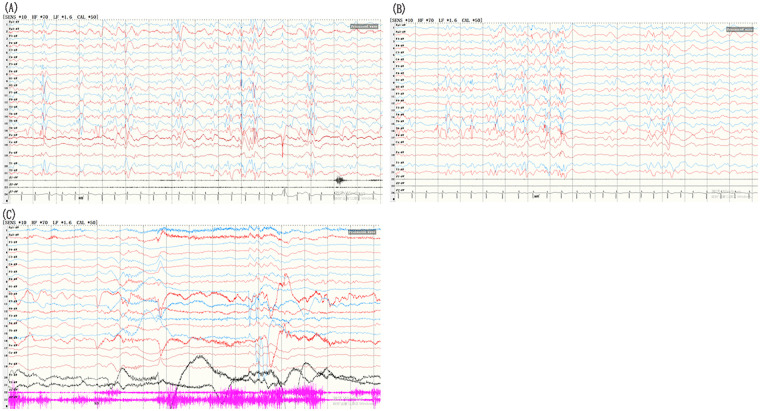
VEEG findings in the patient at 7 months. **(A,B)**VEEG showed hypsarrhythmia during wakefulness **(A)** and sleep **(B)**. **(C)** Epileptic spastic seizures were monitored.

**Figure 2:**
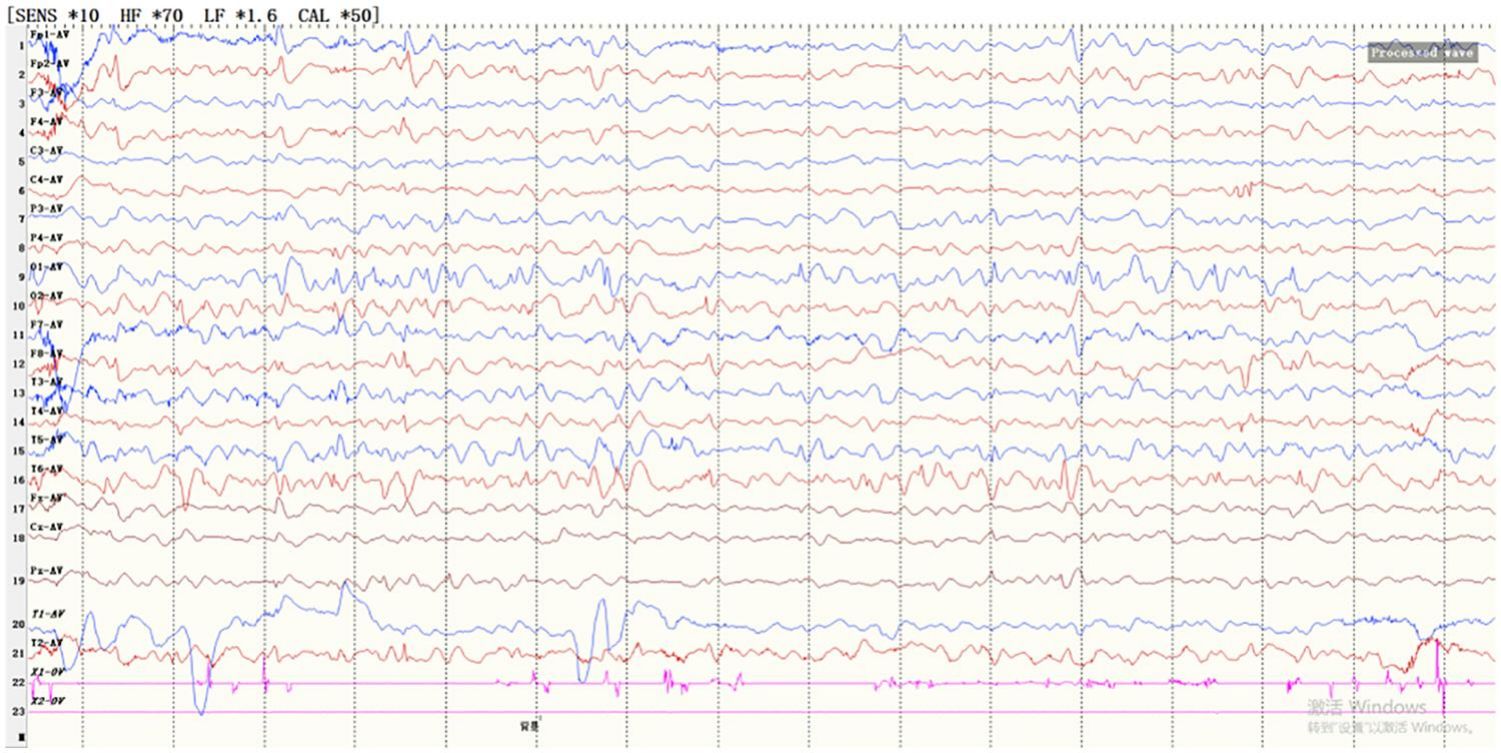
VEEG findings in the patient at 15 months. VEEG showed the presence of spike-slow wave complexes in both the occipital and posterior temporal regions.

The proband was the only child of his parents, he was born at 38 6/7 weeks gestation and weighed 2,600 g at birth. There was no history of perinatal asphyxia, resuscitation, or feeding difficulties after birth. At 6 months of age, he began experiencing epileptic spasms, characterized by episodes of nodding and embracing movements, occurring both in clusters and as isolated events. The patient experienced 2–4 clusters daily, with 1 to 30 spasms per cluster. Each cluster lasted up to 8 min and resolved spontaneously. The seizures occurred exclusively during the drowsy period and upon early awakening. During the interictal period, the infant demonstrated normal alertness and suck/swallow coordination. VEEG revealed hypsarrhythmia and captured multiple spasm episodes (Figure 1). He was treated with adrenocorticotropic hormone (ACTH, 2U × kg^−1^ × d^−1^ × 14d), resulting in a reduction in seizure frequency. Topiramate was subsequently added (10 mg × kg^−1^ × d^−1^), leading to complete control of the spasms. By 15 months of age, VEEG showed improved background activity and the presence of focal spike-and-wave discharges (Figure 2). The patient remained seizure-free for 7 months. Unfortunately, at 16 months of age, epileptic spasms recurred. Clobazam was not effective to control his epilepsy. As of the last follow-up at 2.5 years of age, the child continues to experience several daily spasms, with no other types of epileptic seizures observed.

Since birth, he had failed to achieve any significant developmental milestones. At the first medical consultation at 7 months of age, physical examination disclosed the following: head circumference was 39.5 cm (<−3 SD), weight was 7.8 kg (−1 SD), and length was 67 cm (−1 SD). Neurological examination revealed an inability to fixate or track objects, impaired sound localization, and a diminished social smile response. Vocalizations were sparse. The infant had not achieved head control, rolling, or sitting. Axial hypotonia was noted alongside limb hypertonia. Deep tendon reflexes were normal with flexor plantar responses. Additional findings included bilateral thumb adduction, absent midline hand movements, and no active grasping. By the last follow-up at 2.5 years of age, he showed profound growth failure (head circumference <−3 SD, weight and height <−2 SD), and generalized hypotonia. His clinical course was characterized by global developmental stagnation, despite continuous physical therapy and a 7-month period during which seizures were controlled.

### 3.2 Additional investigation results

**Figure 3:**
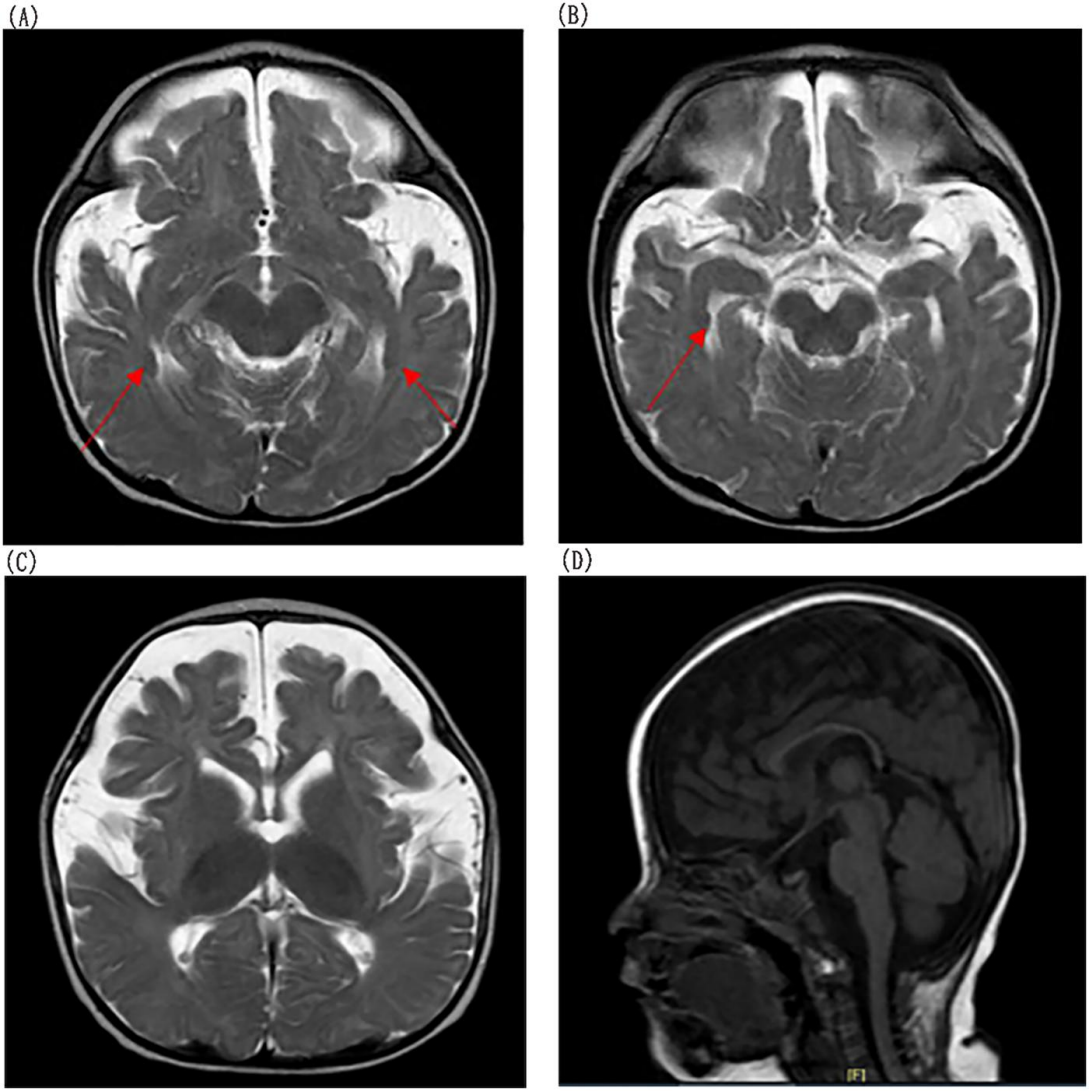
Brain MRI findings. **(A,B)** Periventricular nodular heterotopia (arrowheads) in patient; **(C,D)** Additional findings include small volume in the frontal and temporal lobes, prominent cerebral sulci and widened fissures, ventriculomegaly, and a thin corpus callosum.

**Figure 4:**
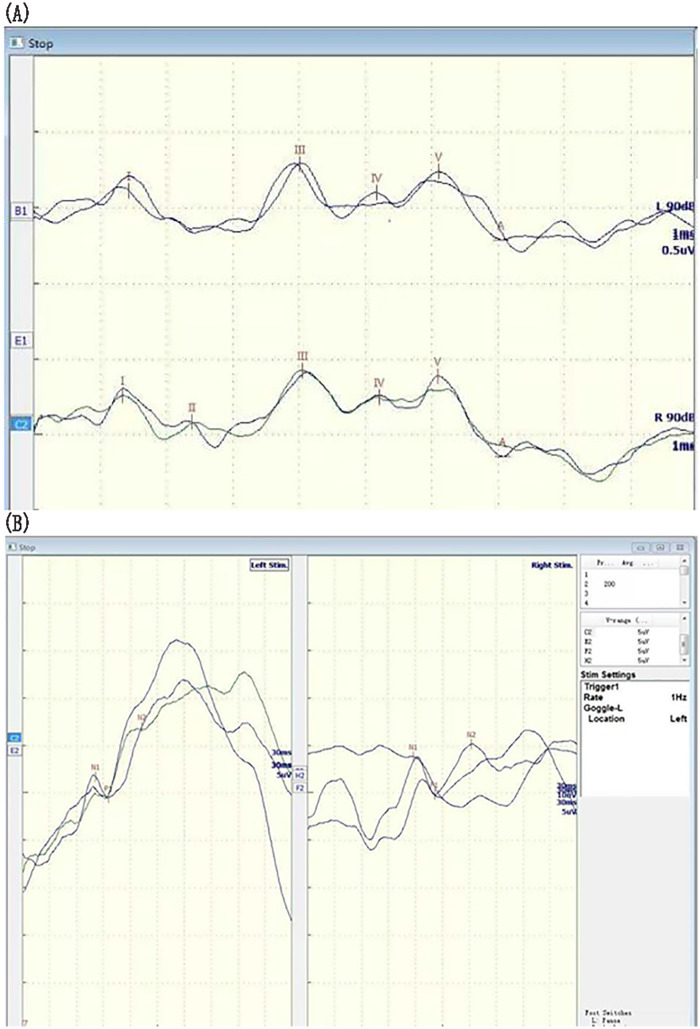
Brainstem auditory evoked potential results. **(A,B)** The traces indicate delayed conduction within the auditory pathways.

Brain MRI revealed PNH, small volume in the frontal and temporal lobes, prominent cerebral sulci and widened fissures, ventriculomegaly and a thin corpus callosum (Figure 3). To assess the level of his development, we used GDS. The GDS revealed he had extremely severe global developmental delay in adaptation (14), large motor movement (18), fine motor movement (11), language (18), and personal-social development (19). Brainstem auditory evoked potentials revealed a delayed auditory conduction pathway (Figure 4). The result of the color Doppler ultrasound of the heart was unremarkable.

### 3.3 Genetic testing

**Figure 5:**
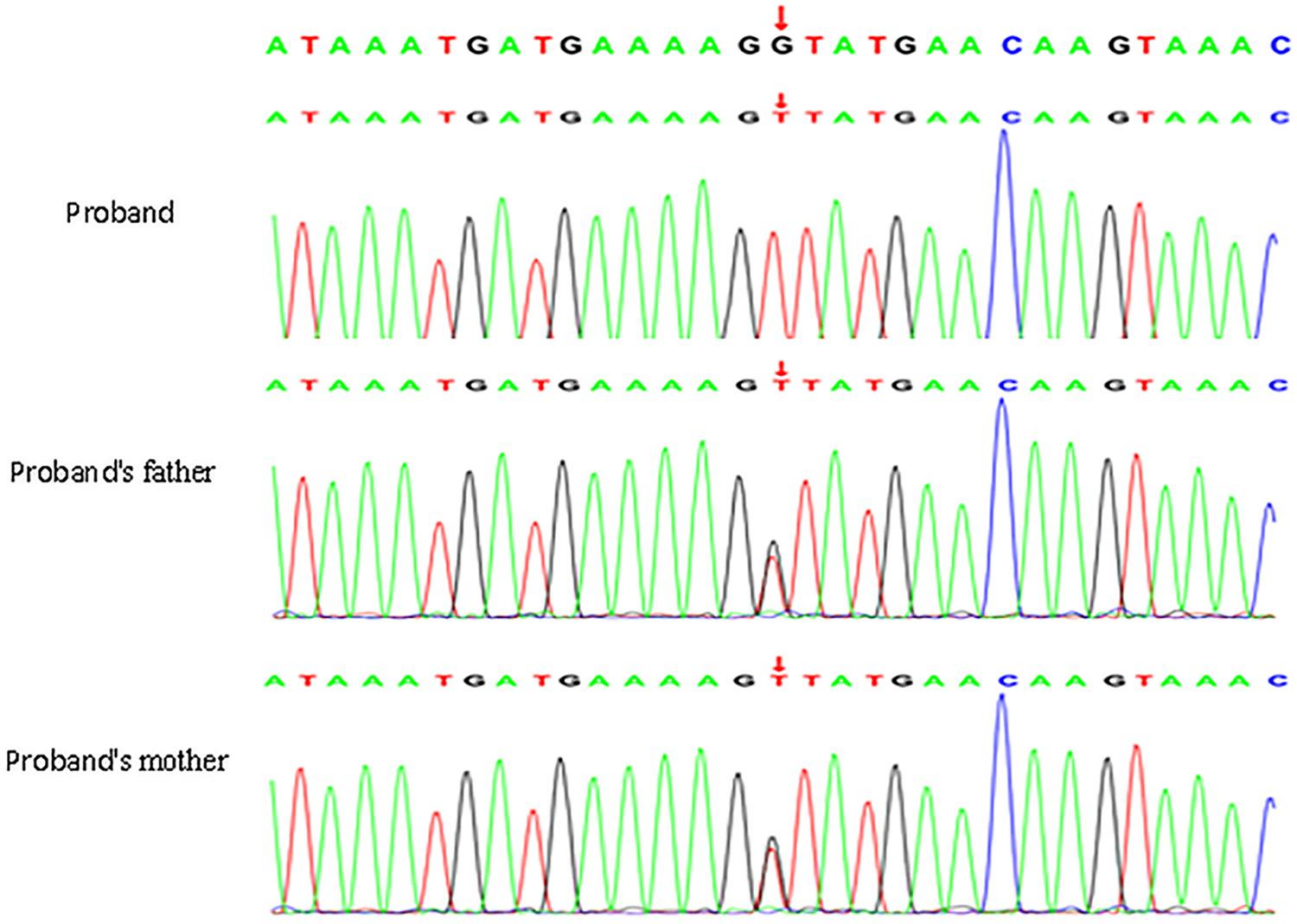
Sanger sequencing chromatograms of the *ARFGEF2* NM_006420.3: c.5181+1G>T variant. The proband is homozygous for the variant, while both parents are heterozygous carriers, confirming autosomal recessive inheritance.

Singleton ES identified a homozygous NM_006420.3: c.5181+1G>T variant in the *ARFGEF2* gene in the proband. Sanger sequencing validation confirmed that both parents were heterozygous carriers of this variant, consistent with an autosomal recessive (AR) inheritance pattern (Figure 5). According to the ACMG standards and guidelines (2019), this variant was classified as Pathogenic (P).

### 3.3 Minigene splicing assay

**Figure 6:**
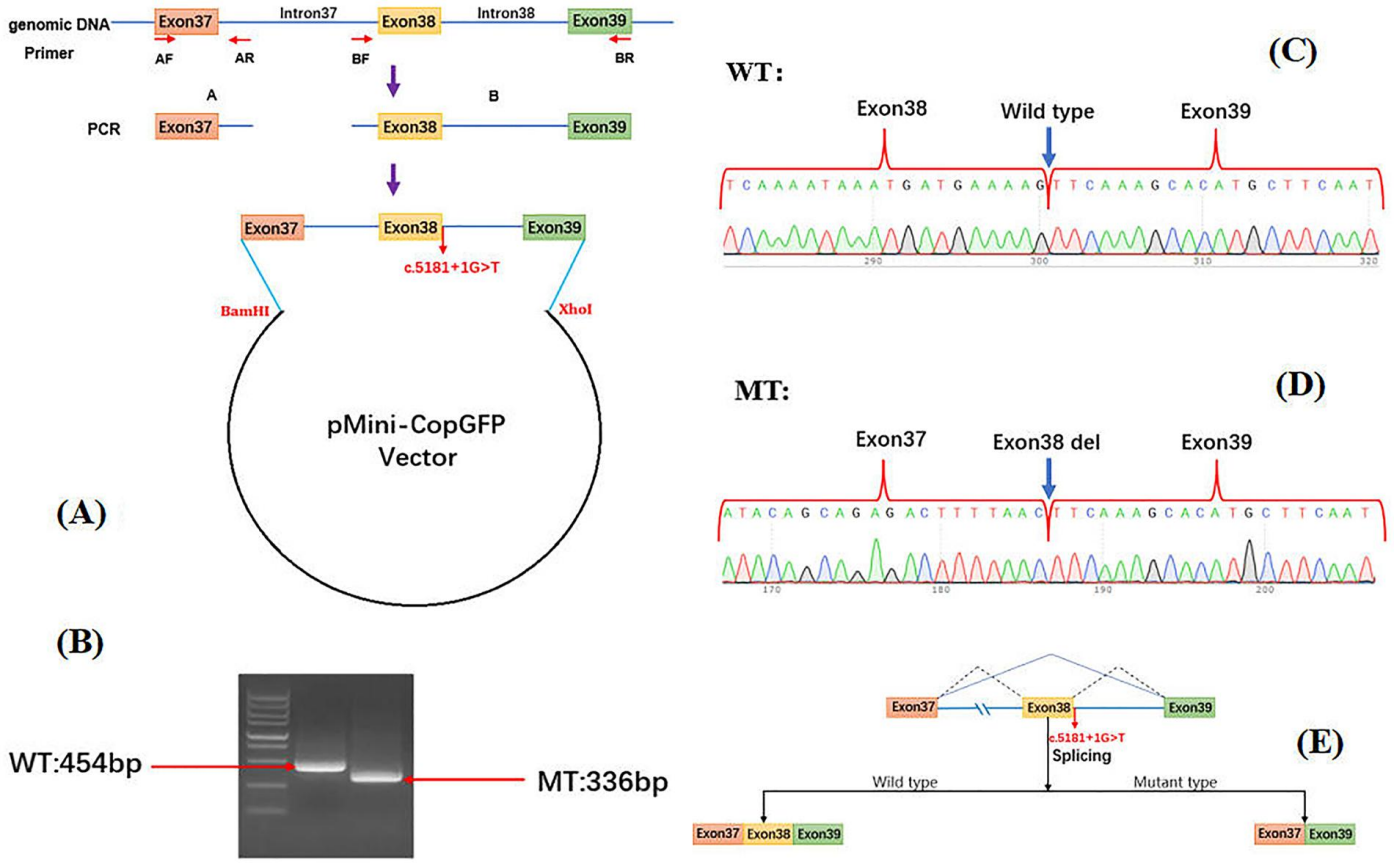
Minigene splicing assay for *ARFGEF2* NM_006420.3: c.5181+1G>T variant and schematic diagram of the splicing pattern. **(A)** Schematic of the wild-type (WT) and mutant (MT) minigene constructs. **(B)** RT-PCR revealed a band for WT and MT. **(C)** WT minigene formed normal mRNA. **(D)** MT plasmid produced a transcript, corresponding to a complete skipping of exon 38. **(E)** Comparison of the splicing patterns between WT and MT.

The wild-type (WT) plasmid produced a 454-bp mRNA transcript, consistent with the expected size, which contained the full sequences of exons 37, 38, and 39. In contrast, the mutant-type (MT) plasmid produced a single 336-bp transcript, corresponding to a complete absence of the 118-bp exon 38. This variant is designated on the mRNA level as NM_006420.3: c.5064_5181del. Translation of this aberrant mRNA is predicted to result in a frameshift and generate a truncated protein, designated as p.Val1689SerfsTer20. In conclusion, these results demonstrate that the NM_006420.3: c.5181+1G>T mutation leads to the complete skipping of exon 38, resulting in a 118-bp deletion that induces a frameshift alteration (Figure 6).

## 4 Discussion

The development of the human cerebral cortex is a highly intricate and dynamic process that follows a precise spatiotemporal sequence, encompassing proliferation, migration, and differentiation. Neural stem cells in the ventricular zone (VZ) and subventricular zone (SVZ) undergo extensive proliferation to generate vast numbers of neurons and glia. Neurons subsequently migrate from their sites of origin in the ventricular regions to populate the cortical plate, establishing the layered architecture of the cortex. Finally, upon reaching their designated positions, neurons undergo structural and functional specialization through axonal and dendritic outgrowth, synaptogenesis, and the assembly of neural circuits, ultimately forming specific architectural patterns (4–6). Malformations of cortical development (MCDs) arise from disruptions in these neurodevelopmental processes and are a major cause of epilepsy and developmental disorders. PNH is one of the most common MCDs. Pathogenic variants in *FLNA* represent the most frequent genetic cause of PNH. In contrast, variants in *ARFGEF2* are associated with autosomal recessive ARPHM, a rarer condition (7, 8).

Vesicular Golgi trafficking is an essential molecular transport process in cells, especially for proper neural development. The Golgi apparatus consists of three main compartments: the cis-Golgi network, medial-Golgi network, and TGN. Within these compartments, vesicular trafficking mechanisms are responsible for the proper distribution and modification of proteins and lipids, which are crucial for the development of neural and radial glial progenitor cells. During neural development, Golgi vesicular trafficking regulates the polarity of neural progenitor cells, which determines their migration paths and destinations. Specifically, in the proliferative periventricular zone, neural progenitor cells rely on specific molecular cues to ensure accurate migration to the cortex. Any disruption in this process can lead to abnormal cortical development and cause a range of neurodevelopmental disorders, including intellectual disabilities and motor impairments. Moreover, Golgi vesicular trafficking plays an essential role in cellular energy metabolism, signal transduction, membrane formation and renewal, and axon guidance. Through vesicular transport, neural progenitor cells are supplied with essential lipids and carbohydrates that form the cell membrane, myelin, and synaptic structures. For example, the synthesis of sphingolipids and glycolipids is critical for the normal function of the nervous system, and this process is regulated within the Golgi apparatus. Disruptions in vesicular trafficking can lead to insufficient production of these crucial molecules, impairing the growth and connectivity of neurons. This process is tightly regulated and modulated in coordination with the endoplasmic reticulum. Specifically, the vesicular trafficking mediated by the Golgi apparatus is essential for proper nerve cell multiplication, migration, cortical assembly, and myelin formation—all of which are fundamental processes for normal brain development and neuronal function (9, 10).

The *ARFGEF2* gene maps to the human chromosome 20q13.13 and encodes the protein BIG2. BIG2 is a large, soluble cytosolic protein (202 kDa) which consists of 1,785 amino acids and contains a highly conserved Sec7 domain. BIG2 is primarily distributed in the trans-Golgi network (TGN) and can also be expressed on endosomes, neuronal axons, and synaptic membranes. Clinically, normal *ARFGEF2* activity is essential for human brain development, as it maintains Golgi-dependent vesicular sorting and neural progenitor polarity by regulating ARF1 activation and COPI vesicle biogenesis. Loss-of-function (LOF) mutations in *ARFGEF2* are mostly autosomal recessive, including nonsense, frameshift, and Sec7 domain-targeted missense variants, which cause severe brain malformations such as microcephaly and PNH (3, 4, 11–13).

These LOF mutations define a subclass of coatopathies, a group of inherited disorders caused by defects in vesicular coat complexes. Vesicular coat complexes are supramolecular assemblies on the cytosolic side of cell membranes, including COPI, COPII, AP complexes, and retromer, which facilitate cargo sorting and intracellular transport. Defects in these complexes impair intracellular transport, leading to developmental abnormalities, among which neurodevelopmental disorders are most common due to the high sensitivity of neural cells to transport disruptions. Thus, *ARFGEF2* LOF mutations link impaired Golgi trafficking, disrupted neural polarity, and congenital brain anomalies. The pathogenic mechanism of these LOF mutations involves disrupting the ARF1-dependent vesicular trafficking pathway. Mutations inactivate, truncate, or degrade BIG2, abolishing its GEF catalytic activity and reducing active ARF1-GTP levels. This impairs the recruitment of COPI, AP-1, AP-3, and other complexes to the TGN and endosomal membranes, disrupting vesicle budding, cargo sorting, and TGN. Neural cells, with their specialized elongated structures, are particularly sensitive to this disruption, leading to abnormal polarity, impaired asymmetric division, and arrested neuronal migration—key factors in PNH development. Disrupted vesicular transport and autophagic flux further induce axonal swelling, neurodegeneration, and impaired brain development, presenting clinically as microcephaly, delayed myelination, and epilepsy. Collectively, these defects form a cascade in the ARF1-coat complex pathway, resulting in severe neurodevelopmental abnormalities, and highlighting the pivotal role of *ARFGEF2* in brain development (10, 13).Animal experiments have confirmed the critical role of BIG2 in neural development. The Arfgef2-mRNA of mice is widely distributed throughout the embryonic central nervous system (CNS), with a slightly higher level observed in the cerebral cortex and diencephalic ventricles. During cortical development, its expression gradually increases from embryonic day 11 (E11) to E17, a period corresponding precisely to the proliferative and migratory phases of the cortex. In contrast, *Arfgef2⁻/⁻* mice exhibited no detectable BIG2 expression in the nervous system and developed periventricular nodular heterotopia (PNH) of varying severity. This distribution pattern implies a role for BIG2 in neural stem cell proliferation and neuronal migration, potentially explaining the pathogenesis of microcephaly and PNH. Furthermore, BIG2 immunoreactivity is localized to the Golgi apparatus in mouse neuroepithelial cells, consistent with its established function in vesicular transport. Collectively, these findings underscore the critical role of ARFGEF2-encoded BIG2 in cortical development (4, 14, 15).

In this study, the patient presented with WS, PNH, microcephaly, and severe developmental delay—symptoms that are likely attributed to vesicular trafficking defects induced by an *ARFGEF2* mutation. We hypothesize that *ARFGEF2* deficiency disrupts the polarity of neural progenitor cells, thereby preventing their proper migration to the cortex and resulting in cortical developmental abnormalities. Additionally, *ARFGEF2* defects may impair the synthesis of glycolipids and sphingolipids, which in turn disrupts neural energy metabolism and membrane stability, further hindering neuronal growth, migration, and synaptogenesis, and ultimately contributing to brain malformations.

West syndrome (WS) is atype of epileptic encephalopathy in infancy. The classic triad includes epileptic spasms, hypsarrhythmia, and developmental stagnation or regression. In addition to epileptic spasms, developmental problems of patients have also been given special attention. The prognosis of WS is highly correlated with its etiology. The etiology of 60% to 70% of WS patients can be determined, which can be classified into acquired and congenital types. The congenital patients are mainly genetic. A genetic etiology can be defined in up to 41% of cases. Genetic explanations have been identified, and among others, include *CDKL5*, *STXBP1*, *IQSEC2*, *ARX*, *TSC1*, *TSC2*, *FOXG1*, and many others. Some chromosomal abnormalities or copy number variants have been associated with WS (16).The proband in this report exhibited global developmental delay from birth. At six months of age, the onset of recurrent epileptic spasms prompted medical consultation. An EEG revealed hypsarrhythmia, leading to a diagnosis of WS. Clinical evaluation revealed severe developmental delay and microcephaly. Brain MRI demonstrated periventricular nodular heterotopia and other cortical developmental malformations, suggesting a genetic etiology. Subsequent ES identified a homozygous pathogenic variant in the *ARFGEF2* gene (NM_006420.3: c.5181+1G>T), leading to a definitive diagnosis of ARPHM. Banne (17) described five patients with WS from a consanguineous family. These patients shared a common phenotype and paraclinical findings. They experienced epileptic spasms at 3 to 9 months of age with hypsarrhythmia pattern on EEG, and evolved into Lennox-Gastaut syndrome (LGS) over time. All five had microcephaly, severe developmental delay and severe hypotonia. The developmental delay occurred earlier than epileptic seizures, and the ultimate developmental outcome was poor. The cranial MRI revealed PNH and a thin corpus callosum. A homozygous variant of c.1958+1G>A in the *ARFGEF2* gene was identified. Apart from this specific variant, our patient's clinical presentation is highly consistent with the reported cases.

Pathogenic *ARFGEF2* variants causing ARPHM are exceedingly rare, with only 22 patients reported to date (Tables 1, 2) (3, 17–24). After excluding two cases with incomplete clinical data, we summarized the characteristics of 21 patients, including our case. The cohort comprised 11 males and 10 females, indicating no gender predominance. Severe global developmental delay, microcephaly (head circumference from −2 to −5 SD), and PNH were uniformly present. Profound motor impairment was universal; only one patient could sit with support at age 7, while most never achieved head control or rolling over. Severe intellectual disability, poor eye contact, and impaired visual tracking and sound localization were common. Dystonia was a prominent feature. All patients exhibited axial hypotonia. Among them, 12 cases concurrently had limb hypotonia, three had limb hypertonia, and three showed variable tone. Two patients had a movement disorder with choreoathetosis.

**Table 1:** Clinical information of patients reported previously.

Case	References	Origin	Gender	Epilepsy	Develop-mental delay	Axial hypotonia	Quadriplegia	Limb dystonia	Chorea	Microcephaly	Feeding difficulties	Growth retardation	Other extraneurological anomalies	Age at death
1	Sheen et al. (3)	Turkish	Female	Absent	Severe	Present	Present	n/a	n/a	Present	Present	Present	n/a	n/a
2	Sheen et al. (3)	Turkish	Male	Absent	Severe	Present	Present	n/a	n/a	Present	Present	Present	n/a	n/a
3	Sheen et al. (3)	Turkish	Female	early-onset refractory epilepsy	Severe	Present	Present	n/a	n/a	<−2SD	n/a	n/a	Absent	n/a
4	Sheen et al. (3)	Turkish	Male	early-onset refractory epilepsy	Severe	Present	Present	n/a	n/a	<−2SD	n/a	n/a	Absent	13Y
5	de Wit et al. (19)	Dutch	Female	Absent	Severe	Present	Present	Dystonic spastic paraplegia	Present	<−2.5SD	Present, gastrostomy	Present	n/a	n/a
6	Tanyalçin et al. (18)	Turkish	Male	Absent	Severe	Present	Present	hypotonia	n/a	−3.6SD	Present, gastrostomy and tracheostomy	Present	Obstructive cardiomyopathy, recurrent respiratory infections	12Y9M
7	Tanyalçin et al. (18)	Turkish	Female	Absent	Severe	Present	Present	hypotonia	n/a	−2.4SD	Present, gastrostomy	Present	Absent	9Y
8	Tanyalçin et al. (18)	Turkish	Male	focal tonic seizures	Severe	Present	Present	hypotonia	n/a	−3.4SD	Present	Present	Facial dysmorphism, complicated respiratory infection	5Y11M
9	Banne et al. (17)	Palestinian	Male	WS evolved into LGS	Severe	Present	Present	hypotonia	n/a	−3SD to −5SD	n/a	n/a	Absent	n/a
10	Banne et al. (17)	Palestinian	Male	WS evolved into LGS	Severe	Present	Present	hypotonia	n/a	−3SD to −5SD	n/a	n/a	Absent	n/a
11	Banne et al. (17)	Palestinian	Male	WS evolved into LGS	Severe	Present	Present	hypotonia	n/a	−3SD to −5SD	n/a	n/a	Absent	n/a
12	Banne et al. (17)	Palestinian	Female	WS evolved into LGS	Severe	Present	Present	hypotonia	n/a	−3SD to −5SD	n/a	n/a	Absent	n/a
13	Banne et al. (17)	Palestinian	Male	WS evolved into LGS	Severe	Present	Present	hypotonia	n/a	−3SD to −5SD	n/a	n/a	Absent	n/a
14	Bardón-Cancho et al. (20)	n/a	Female	myoclonic seizures	Severe	Present	Present	alternating low axial tone with jerking movements of the extremities exacerbated by the emotions	n/a	−2SD	Absent	Present	n/a	n/a
15	Bardón-Cancho et al. (20)	n/a	Male	Absent	Severe	Present	Present	axial hypotonia with fluctuating limbs tone, dystonic-athetoid	n/a	−3SD	Absent	Present	n/a	n/a
16	Yilmaz et al. (21)	n/a	Female	Absent	Severe	Present	Present	hypotonia	Present	<−2SD	Present	Present	Left ventricle noncompaction cardiomyopathy, red hair, strabismus	n/a
17	Neghery et al. (22)	Saudi	Female	GTCS, clonic seizures	Severe	Present	Present	spasticity in all extremities	n/a	Present	n/a	Present	n/a	n/a
18	Neghery et al. (22)	Saudi	Male	myoclonic seizures	Severe	Present	Present	spasticity in all extremities	n/a	Present	n/a	Present	n/a	n/a
19	Alojair et al. (23)	Saudi	Female	GTCS	Severe	Present	Present	hypotonia	n/a	<−4SD	Present	Present	n/a	n/a
20	Alojair et al. (23)	Saudi	Female	GTCS	Severe	Present	Present	hypotonia	n/a	<−4SD	n/a	Present	n/a	n/a
21	Our case	China	Male	epileptic spasm	Severe	Present	Present	hypotonia	Absent	<−3SD	Absent	Present	Absent	n/a

n/a, not available.

**Table 2:** Genetic and auxiliary examination results of patients reported previously.

Case	cDNA change	Mutation Type	Protein change	Exon	zygote	MRI findings	VEEG
1	c.625G>A	Missense	Glu209Lys	6	homozygote	PNH, cortical atrophy, putaminal hyperintensity, hyperintensity in putamen	Slow background, paroxysmal *θ* activity
2	c.625G>A	Missense	Glu209Lys	6	homozygote	PNH, cortical atrophy, myelination delayed, thin corpus callosum	Background dysrhythmia with occasional spike and wave activity
3	c.242_249delins7	Missense	Pro81Glnfs*34	3	compound heterozygote	PNH, cortical atrophy, white matter hyperintensity, ventricles are prominent or enlarged, thin corpus callosum, ventricular dilation	Hypsarrhythmia
4	c.242_249delins7	Missense	Pro81Glnfs*34	3	compound heterozygote	PNH, cortical atrophy	Hypsarrhythmia
5	c.2031_2038dup and c.3798_3802del	Frameshift	Gln680Profs*2 and Phe1267Glyfs*17	15	compound heterozygote	PNH, myelination delayed, generalized atrophy	Diffuse *δ* activity
6	c.242_249delins7	Missense	Pro81Glnfs*34	3	compound heterozygote	PNH, cortical atrophy, paucity of the white matter, hyperintensity in putamen, hippocampus atrophy, thin corpus callosum	Diffuse slowing
7	c.242_249delins7	Missense	(Pro81Glnfs*34	3	compound heterozygote	PNH, cortical atrophy and paucity of the white matter, thin corpus callosum, hippocampus atrophy	Slow background
8	c.242_249delins7	Missense	Pro81Glnfs*34	3	compound heterozygote	PNH, cortical atrophy, paucity of the white matter, myelination delayed, hyperintensity in putamen, hippocampus atrophy, thin and short corpus callosum	Epileptiform
9	c.1958(+1) G>A	Splicing	/	15	homozygote	PNH, thin corpus callosum	Hypsarrhythmia
10	c.1958(+1) G>A	Splicing	/	15	homozygote	PNH, thin corpus callosum	Hypsarrhythmia
11	c.1958(+1) G>A	Splicing	/	15	homozygote	PNH, thin corpus callosum	Hypsarrhythmia
12	c.1958(+1) G>A	Splicing	/	15	homozygote	PNH, thin corpus callosum	Hypsarrhythmia
13	c.1958(+1) G>A	Splicing	/	15	homozygote	PNH, thin corpus callosum	Hypsarrhythmia
14	c.388C>T	Nonsense	Gln130*	4	homozygote	PNH, thin corpus callosum, ventriculomegaly, hyperintensity in putamen and thalamus, small lenticular nucleus	Slow background with diffuse epileptiform activity, myoclonic
15	c.388C>T	Nonsense	Gln130*	4	homozygote	PNH, thin corpus callosum, ventriculomegaly, hyperintensity in putamen, small lenticular nucleus	n/a
16	c. 5126 G>A	Nonsense	Trp1709*	38	homozygote	PNH, cortical atrophy, hyperintensity in putamen and caudate nucleus	Normal
17	c.3974G>A	Nonsense	Trp1325*	29	homozygote	PNH, hyperintensity in putamen and globus pallidus	n/a
18	c.3974G>A	Nonsense	Trp1325*	29	homozygote	n/a	n/a
19	c.1958(+1) G>A	Splicing	/	15	homozygote	PNH, hyperintensity in putamen, thin corpus callosum, ventriculomegaly, paucity of the white matter	Sharp waves over the right occipital area
20	c.1958(+1) G>A	Splicing	/	15	homozygote	PNH, hyperintensity in putamen, thin corpus callosum, ventriculomegaly, paucity of the white matter	n/a
21	c.5181+1 G>T	Splicing	Val1689SerfsTer20	38	homozygote	PNH, cortical atrophy, ventriculomegaly, thin corpus callosum	Hypsarrhythmia

n/a, not available.

The incidence of epilepsy in this disorder is high, affecting 66.7% (14/21) of patients. Seizure onset was early, often refractory to medication, and occurred before 2 years of age in all affected individuals, with 13 cases starting within the first year of life. Six patients, including ours, presented with WS. Five patients from a single pedigree eventually evolved into LGS. Seizure types were diverse: two patients had only generalized tonic-clonic seizures (GTCS), one had focal tonic seizures, two had myoclonic seizures, one had both myoclonic and GTCS, and two were described merely as early refractory epilepsy without further specification. Previous reports provided limited details on antiseizure medication regimens. Our patient was treated with ACTH and topiramate, which controlled the epileptic spasms for seven months. However, with increasing age, seizures recurred. The combination of topiramate and clobazam proved ineffective, and no further seizure control was achieved thereafter. The parents declined trials of other treatment options, so the potential efficacy of these alternatives remains uncertain. Extra-neurological manifestations were also noted. Fourteen patients had documented failure to thrive, among whom nine had feeding difficulties. Three required gastrostomy, yet their growth remained subnormal. Two patients were diagnosed with cardiomyopathy, and one had a history of recurrent infections. No significant endocrine disorders were reported.

All patients underwent brain MRI, which confirmed that periventricular nodular heterotopia was a universal finding, frequently accompanied by other brain malformations. These included callosal dysgenesis (14/21), signal abnormalities in the putamen (8/21), diminished or abnormal white matter signals (6/21), cortical atrophy (10/21), ventriculomegaly (3/21), hippocampal atrophy (3/21), delayed myelination (3/21), signal abnormalities in the caudate nucleus and globus pallidus (3/21), and small lentiform nuclei (2/21). EEG abnormalities predominantly involved background activity disturbances. Hypsarrhythmia was documented in eight cases.

Based on genotypes reported in the literature and a review of the ClinVar database, 28 pathogenic variants in the *ARFGEF2* gene have been identified. These include six large deletions/duplications and three intronic mutations. Among the remaining variants, nonsense mutations were the most common (*n* = 10), followed by frameshift mutations (*n* = 6), with missense mutations being rare (*n* = 2). Sequencing of our proband identified a canonical splice-site mutation (NM_006420.3: c.5181+1G>T).

Collectively, the reported cases indicate a consistently poor prognosis for ARPHM caused by *ARFGEF2* mutations, characterized by drug-resistant epilepsy, a lack of developmental progress even with rehabilitation, and compromised longevity. Of the 21 documented patients, 4 had died by the time of reporting, at ages between 5 years 11 months and 13 years. Our patient's presentation is consistent with this profile, exhibiting uncontrolled seizures and global developmental stagnation at the last follow-up at 2.5 years of age.

## 5 Conclusion

ARPHM resulting from *ARFGEF2* mutations, while exceedingly rare, confers a grave prognosis marked by severe symptoms, profound therapeutic challenges, and premature mortality. This underscores the paramount importance of genetic testing for definitive diagnosis. We report a male child whose clinical presentation was highly consistent with ARPHM. ES identified a homozygous, autosomal recessive *ARFGEF2* variant (NM_006420.3: c.5181+1G>T). A minigene splicing assay demonstrated that this variant causes aberrant splicing and a truncated protein, thereby confirming its pathogenicity. As the first report of this splice-site variant, our study expands the mutational spectrum of *ARFGEF2* in ARPHM and provides new evidence for the critical role of Golgi vesicular trafficking in neural development. These findings further help to define “coatopathies” as a category of developmental brain defects, expanding our understanding of neurodevelopmental disorders and offering new directions for clinical diagnosis and intervention.

## Data Availability

The original contributions presented in the study are included in the article/Supplementary Material, further inquiries can be directed to the corresponding authors.
